# Influence of *Chlorella vulgaris* on growth, digestibility and gut morphology and microbiota of weaned piglet

**DOI:** 10.1038/s41598-022-10059-5

**Published:** 2022-04-09

**Authors:** Cátia F. Martins, Paolo Trevisi, Diogo F. Coelho, Federico Correa, David M. Ribeiro, Cristina M. Alfaia, Mário Pinho, José M. Pestana, Miguel P. Mourato, André M. Almeida, Carlos M. G. A. Fontes, João P. B. Freire, José A. M. Prates

**Affiliations:** 1 CIISA-Centre for Interdisciplinary Research in Animal Health, Faculty of Veterinary Medicine, University of Lisbon, Lisbon, Portugal; 2 LEAF-Linking Landscape, Environment, Agriculture and Food, Higher Institute of Agronomy, University of Lisbon, Lisbon, Portugal; 3 DISTAL-Department of Agricultural and Food Sciences, University of Bologna, Bologna, Italy; 4 NZYTech-Genes and Enzymes, Estrada do Paço do Lumiar, Campus do Lumiar, Edifício E, 1649-038 Lisbon, Portugal

**Keywords:** Zoology, Animal physiology

## Abstract

The purpose of this study was to evaluate the impact of *Chlorella vulgaris* (5% in the diet), supplemented or not with two exogenous carbohydrase mixtures on piglets’ performance, nutrient digestibility and gut morphology, fermentation products and microbiota. Forty-four male piglets weaned at 28 days of age, with 11.2 ± 0.46 kg of live weight, were used and assigned to 1 of 4 dietary treatments: cereal and soybean meal based-diet (control, n = 11), control diet with 5% of *C. vulgaris* (CH, n = 10), CH diet supplemented with 0.005% of Rovabio^®^ Excel AP (CH + R, n = 10) and CH diet supplemented with 0.01% of a recombinant 4-carbohydrase mixture (CH + M, n = 11). Growth performance was not changed by the of *C. vulgaris* inclusion during 21 days of trial. However, total tract apparent digestibility of nutritional fractions was negatively impacted by the inclusion. In addition, the viscosity of duodenum plus jejunum contents slightly increased in all groups fed with the microalga. In contrast, dietary microalga increased duodenum villus height and promoted a healthier gut microbiota, with higher abundance of some specific bacterial taxa (*Colidextribacter*, *Oscillospira* and *Lactobacillus*). This study indicates that the dietary inclusion of 5% *C. vulgaris* improves piglets’ gut health without impairing performance. Data also indicate that *C. vulgaris* reduces nutrient digestibility but promotes compensatory developments of gut mucosa and prebiotic effects. Dietary supplementation with exogenous carbohydrases does not seem to be necessary for this inclusion level. Therefore, the incorporation of CH as a sustainable feed ingredient in piglets’ nutrition is a viable alternative approach.

## Introduction

The post-weaning period is one of the most critical periods in swine production. It is associated to social, environmental and nutritional changes. In addition, piglets’ immune system is not yet fully developed and, therefore, animals are more susceptible to several digestive and respiratory pathologies. Also, recently weaned pigs experience strong structural and physiological changes in the intestine^1^. Furthermore, the use of antibiotics for preventive or therapeutic purposes has been associated with an increased occurrence of antimicrobial resistant microorganisms, showing that strategies to reduce or prevent their utilization are necessary.

Among these strategies, several feed-based solutions are considered interesting alternatives. Innovative compounds and feedstuffs, like microalgae, are of interest for their prebiotic properties in order to cope with post-weaning stress^2^. In addition, they are considered sustainable feedstuffs that do not compete for land and other resources necessary to produce food for human consumption^3^. Furthermore, they have the potential to fixate carbon dioxide from the atmosphere, thus contributing to mitigate global warming. Furbeyre et al*.*^4^ observed that a 1% dietary inclusion of Spirulina and *Chlorella vulgaris* as an alternative to antibiotics improved the intestinal health in weaned piglets. As mentioned in the literature, small inclusion levels of different microalgae in piglet diets increase gut health, albeit more research is needed in order to understand, establish and validate their effect on the intestinal microflora^3^.

Although microalgae have a high nutritive value and are an interesting sustainable alternative to cereals and soybean in swine diets, the particular characteristics of their recalcitrant cell wall make them rather indigestible for monogastric animals^5^. However, enzymes that degrade the cell wall, like Carbohydrate-active enzymes (CAZymes or carbohydrases), might improve their utilization with positive effects on nutrient bioavailability, in addition to promote the prebiotic properties of the insoluble polysaccharides typical of these matrices. Accordingly, Coelho et al*.*^6^ described a 4-CAZyme mixture able to degrade in vitro the *C. vulgaris* cell wall. Furthermore, Martins et al*.*^7^ recently studied a higher level of dietary Spirulina incorporation (10% dietary inclusion), either individually and in combination with 2 commercial carbohydrases, in post-weaned piglets’ diets. Authors showed that lysozyme is efficient in the degradation of this microalga cell wall in the piglet’s gut.

Accordingly, the aim of this work was to evaluate the impact of 5% *C. vulgaris* in the diet, combined with 2 exogenous carbohydrase mixtures (Rovabio® Excel AP and the 4-carbohydrase mixture tested by Coelho et al*.*^6^, on piglets’ performance, nutrient digestibility and gut morphology, fermentation products and microbiota profile.

## Results

### Growth performance

The effect of experimental diets on growth performance of piglets are presented in Table 1. The piglets’ weight was similar among experimental groups at the beginning and end of the trial (P > 0.05), with mean values of 11.2 and 23.1 kg, respectively. However, the control group had a lower average daily feed intake (ADFI), with 87 g/day lower feed intake by comparison with the CH-fed groups. In contrast, average daily weight gain (ADG) and feed conversion ratio (FCR) were similar for all experimental groups (P > 0.05).

**Table 1:** Effect of diets on feed intake and growth performance of piglets.

	Diets				SEM	P-value
	Control	CH	CH + R	CH + M		
Initial weight, kg	11.1	11.1	11.3	11.2	0.105	0.851
Final weight, kg	22.3	23.3	23.5	23.1	0.247	0.349
ADFI, g	768^a^	852^b^	857^b^	856^b^	11.6	0.008
ADG, g	535	581	579	569	8.5	0.188
FCR	1.44	1.47	1.48	1.51	0.013	0.282

Control = control diet; CH = *Chlorella vulgaris* diet; CH + R = *C. vulgaris* diet supplemented with Rovabio® Excel AP; CH + M = *C. vulgaris* diet supplemented with a pre-selected enzymatic mixture.

*ADFI* average daily feed intake, *ADG* average daily gain, *FCR* feed conversion ratio.

^a,b^Values within a row with different superscripts differ significantly at P < 0.05.

### Digestibility of nutrients

The effect of diets on total tract apparent digestibility (TTAD) of nutrients and consistency of faeces are shown in Table 2. The experimental treatments affected all TTAD nutritional fractions, with the exception of crude fat (CF). TTAD of dry mater (DM) and organic matter (OM) were significantly higher in control group, with an average difference of 2.5 and 2.1 percentage points, respectively, compared with the other groups. For these parameters, the CH and CH + M groups had the lowest values, while the CH + R group had the intermediate value, by comparison with the control group. Groups fed with microalga, supplemented or nor with exogenous enzymes, had a decrease in TTAD of crude protein (CP) in about 4.5 percentage points (P < 0.0001) in comparison with the control group. Regarding this last-mentioned parameter, the CH group had the lowest value compared with the control and CH + R groups.

**Table 2:** Effect of diets on total tract apparent digestibility (TTAD) of nutrients and consistency of faeces.

	Diets				Period		SEM	P-value*	
	Control	CH	CH + R	CH + M	1st	2nd		Diet	Period
**TTAD, %**									
DM	89.8^a^	86.8^b^	88.1^c^	86.9^b^	88.2	87.6	0.25	< 0.0001	0.0019
OM	90.0^a^	87.4^b^	88.5^c^	87.7^b^	88.7	88.1	0.22	< 0.0001	0.0029
CP	86.4^a^	81.3^b^	82.9^c^	81.6^bc^	83.4	82.6	0.46	< 0.0001	0.0193
CF	78.8	77.1	78.7	77.9	78.4	77.8	0.38	0.2091	0.0827
NDF	75.9^a^	68.0^b^	72.0^c^	63.9^d^	70.7	69.2	0.79	< 0.0001	0.0165
ADF	50.8^a^	32.3^b^	39.3^c^	35.0^d^	40.1	38.6	1.42	< 0.0001	0.2506
Hemicellulose	82.7^a^	77.1^b^	80.3^c^	73.5^d^	79.0	77.8	0.63	< 0.0001	0.0060
Cellulose	35.4^ab^	34.1^a^	39.6^b^	40.4^b^	38.7	36.1	1.16	0.0518	0.0467
Faecal score^1^	0.560	0.950	1.00	1.13	0.85	0.97	0.074	0.1849	0.1165

Control = control diet; CH = *Chlorella vulgaris* diet; CH + R = *C. vulgaris* diet supplemented with Rovabio® Excel AP; CH + M = *C. vulgaris* diet supplemented with a pre-selected enzymatic mixture.

*DM* dry matter, *OM* organic matter, *CP* crude protein, *CF* crude fat, *NDF* neutral detergent fibre, *ADF* acid detergent fibre.

^1^Faecal score: 0 (normal), 1 (soft faeces) or 2 (diarrhoea).

*The interaction of the 2 factors (diet × period) was not significant for all the variables.

^a,b,c,d^Values within a row with different superscripts differ significantly at P < 0.05.

TTAD values of neutral detergent fibre (NDF), acid detergent fibre (ADF) and hemicellulose were significantly different between all experimental groups, with the control group showing the highest values, followed by the CH + R group, whereas the CH + M group had the lowest value of NDF and hemicellulose, and the CH group of ADF. TTAD of cellulose was higher in the CH + M group, albeit with no significant differences in comparison with the CH + R group. It showed, however, significant differences when compared with the CH group (6.3 percentage points increase). When we look at the results considering the 2 different collection periods, there were significant differences for TTAD for all nutrients, except for CF and ADF. For the second period, the TTAD results were lower than those of the first period.

Concerning faecal scores, no significant differences between the experimental groups were detected, neither considering the effect of diet nor the effect of collecting period (P = 0.1849 and 0.1165, respectively).

### Gut length, content viscosity, pH and histology

In Table 3 we present the effect of diets on gastrointestinal tract variables, relative length, content viscosity and pH and intestinal morphology traits. Diets had no effect on the relative length of small and large intestines (P > 0.05). Digesta viscosity of duodenum plus jejunum was 28% higher in groups fed with the microalga in comparison with the control group. Regarding digesta viscosity, no effect was observed for the ileum content of all dietary treatments (P = 0.6762). The stomach, caecum and colon pH values were similar for all dietary treatments (P > 0.05). In contrast, diet had a significant effect on pH of duodenum plus jejunum and ileum contents (P = 0.0028 and P = 0.0383, respectively). Regarding the pH of duodenum plus jejunum content, no significant differences were found between the CH + M and the CH and CH + R. Additionally, the duodenum plus jejunum content pH for the control group was significantly higher that of the CH group. Groups fed with CH had a 7.1% reduction in the ileum content pH by comparison with the control group.

**Table 3:** Effect of diets on gastrointestinal tract variables and intestinal morphology of piglets.

	Diets				SEM	P-value
	Control	CH	CH + R	CH + M		
**Relative length of gastrointestinal tract, m/kg**						
Small intestine	0.733	0.657	0.656	0.710	0.010	0.0882
Large intestine	0.145	0.139	0.142	0.151	0.003	0.6056
**Content viscosity, cP**						
Duodenum + jejunum	2.64^a^	3.64^b^	3.63^b^	3.70^b^	0.15	0.0202
Ileum	5.00	5.64	4.73	5.42	0.28	0.6762
**pH**						
Stomach	3.95	4.10	4.07	3.86	0.063	0.4475
Duodenum + jejunum	5.67^ab^	5.42^c^	5.57^bc^	5.73^a^	0.033	0.0028
Ileum	6.37^a^	5.93^b^	5.87^b^	5.96^b^	0.069	0.0383
Caecum	5.67	5.71	5.65	5.92	0.055	0.3120
Colon	6.19	6.25	6.19	6.22	0.036	0.9260
**Villus height, μm**						
Duodenum	339^a^	424^b^	414^b^	402^b^	10.8	0.0160
Jejunum	376	428	399	399	17.7	0.7893
Ileum	327	370	376	321	9.7	0.0978
**Villus width, μm**						
Duodenum	187	175	177	192	2.9	0.1293
Jejunum	152	146	159	163	3.6	0.4021
Ileum	195	182	176	186	3.9	0.3696
**Crypt depth, μm**						
Duodenum	503	469	489	431	11.2	0.1130
Jejunum	345	343	367	349	7.0	0.6332
Ileum	313	299	272	267	7.7	0.0902
**Villus height/crypt depth**						
Duodenum	0.686^a^	0.911^b^	0.859^b^	0.967^b^	0.03	0.0088
Jejunum	1.10	1.26	1.10	1.14	0.05	0.6784
Ileum	1.07	1.26	1.44	1.24	0.05	0.0775

Control = control diet; CH = *Chlorella vulgaris* diet; CH + R = *C. vulgaris* diet supplemented with Rovabio® Excel AP; CH + M = *C. vulgaris* diet supplemented with a pre-selected enzymatic mixture.

^a,b,c^Values within a row with different superscripts differ significantly at P < 0.05.

The control group had lower duodenum villus heights when compared with the other groups fed with the microalga. The incorporation of microalga caused an 18% increase by comparison with the control group. Consequently, the villus height to crypt depth ratio were higher for groups fed with microalga (P = 0.0088). The villus height for jejunum and ileum were similar for all experimental treatments. Additionally, for the other 2 variables measured, villus width and crypt depth, at 3 different gut locations, no significant differences were detected (P > 0.05).

### Gut volatile fatty acids

The effect of diets on volatile fatty acids (VFA) concentration of piglets’ caecum and colon contents is shown in Table 4. For VFA concentration in caecum, with the exception of isovaleric acid (iC5), the CH + M group had lower values than those of the other groups, with a reduction of 26%, 30%, 39%, 53% and 31% for acetic acid (C2), propionic acid (C3), butyric acid (C4), valeric acid (C5) and total concentration, respectively. No differences were observed between control, CH and CH + R groups regarding VFA concentration in caecum content.

**Table 4:** Effect of diets on volatile fatty acids (VFA) concentration in caecum and colon of piglets.

Item^1^	Diets				SEM	P-value
	Control	CH	CH + R	CH + M		
**VFA concentration in caecum, mmol/L**						
C2	20.2^a^	19.5^a^	21.4^a^	15.1^b^	0.910	0.0079
C3	13.0^a^	11.0^a^	12.5^a^	8.54^b^	0.624	0.0042
C4	6.17^a^	6.64^a^	7.70^a^	4.18^b^	0.452	0.0114
iC5	0.845	0.144	0.184	0.069	0.177	0.3678
C5	2.05^a^	1.95^a^	1.86^a^	0.920^b^	0.176	0.0444
Total	42.3^a^	39.3^a^	43.7^a^	28.8^b^	1.920	0.0005
**VFA concentration in colon, mmol/L**						
C2	21.2^a^	14.9^bc^	16.2^b^	12.8^c^	0.785	< 0.0001
C3	10.6^a^	8.03^b^	7.98^b^	6.73^c^	0.470	0.0082
C4	5.78^a^	4.32^ab^	4.47^ab^	3.17^b^	0.299	0.0187
iC5	0.777^a^	0.413^b^	0.499^b^	0.326^b^	0.054	0.0030
C5	2.05^a^	1.39^ab^	1.43^ab^	0.880^b^	0.136	0.0100
Total	40.4^a^	29.1^b^	30.6^b^	23.9^b^	1.611	0.0003

Control = control diet; CH = *Chlorella vulgaris* diet; CH + R = *C. vulgaris* diet supplemented with Rovabio® Excel AP; CH + M = *C. vulgaris* diet supplemented with a pre-selected enzymatic mixture.

^1^C2, C3, C4, C5 and iC5 are acetic, propionic, butyric, valeric and isovaleric acids, respectively.

^a,b,c^Values within a row with different superscripts differ significantly at P < 0.05.

For VFA concentration in the colon, there was a significant influence of dietary treatments. By comparison with the control group, microalga-fed experimental groups had a significant decrease of 30%, 24% and 40%, respectively, for the concentration of C2. For the C3 concentration, the same comparisons led a decrease of 24%, 25% and 37%, respectively. For the C4 and C5 concentrations, only the CH + M group had a significant decrease of 45% and 57%, when compared with the control group. For iC5 and total VFA concentrations, comparatively with the control group, all microalga-fed groups had a significant reduction. Regarding the CH vs. CH + R groups’ comparison, similar values were observed for all VFA concentrations in the colon. When the CH group was compared with the CH + M, the only recorded significant difference concerned the C3 concentration that was 16% increase in the CH + M group. When comparing the 2 groups supplemented with enzymes regarding C3 concentration, there was significant 16% increase in the CH + M group.

### Gut microbiota

As a sanity check for the sequencing procedure the rarefaction curve was plotted (please see detail in Supplementary Fig. S1). Overall, all samples reached the plateau point suggesting a good sequencing efficiency and indicating that the sequencing procedure has captured all the taxonomic variability present in that specific ecosystem.

All the reads that were maintained in every step of the bioinformatic analysis are presented in Supplementary Table S1. At the end, all samples had a high number of reads (53.6 in average), that resulted in a total of 1684 different Amplicon Sequence Variants (ASVs). Of these, 99.4% were assigned at Phylum level to 22 different Phyla, with Bacteroidota (Bacteroidetes) 48.6% and Firmicutes 32.3% comprising the majority of Phyla. At family level, a total of 81 families were identified (Prevotellaceae 38.7%, Oscillospiraceae 7.31% and Rikenellaceae 5.52%), and at Genus level 176 genera (Prevotella 21.4%, Rikenellaceae_RC9_gut_group 4.95% and *Alloprevotella* 4.30%). Composition plots showing the relative abundance of the top 10 taxa for Phylum, Family and Genus are reported in Supplementary Fig. S2.

**Figure 1:**
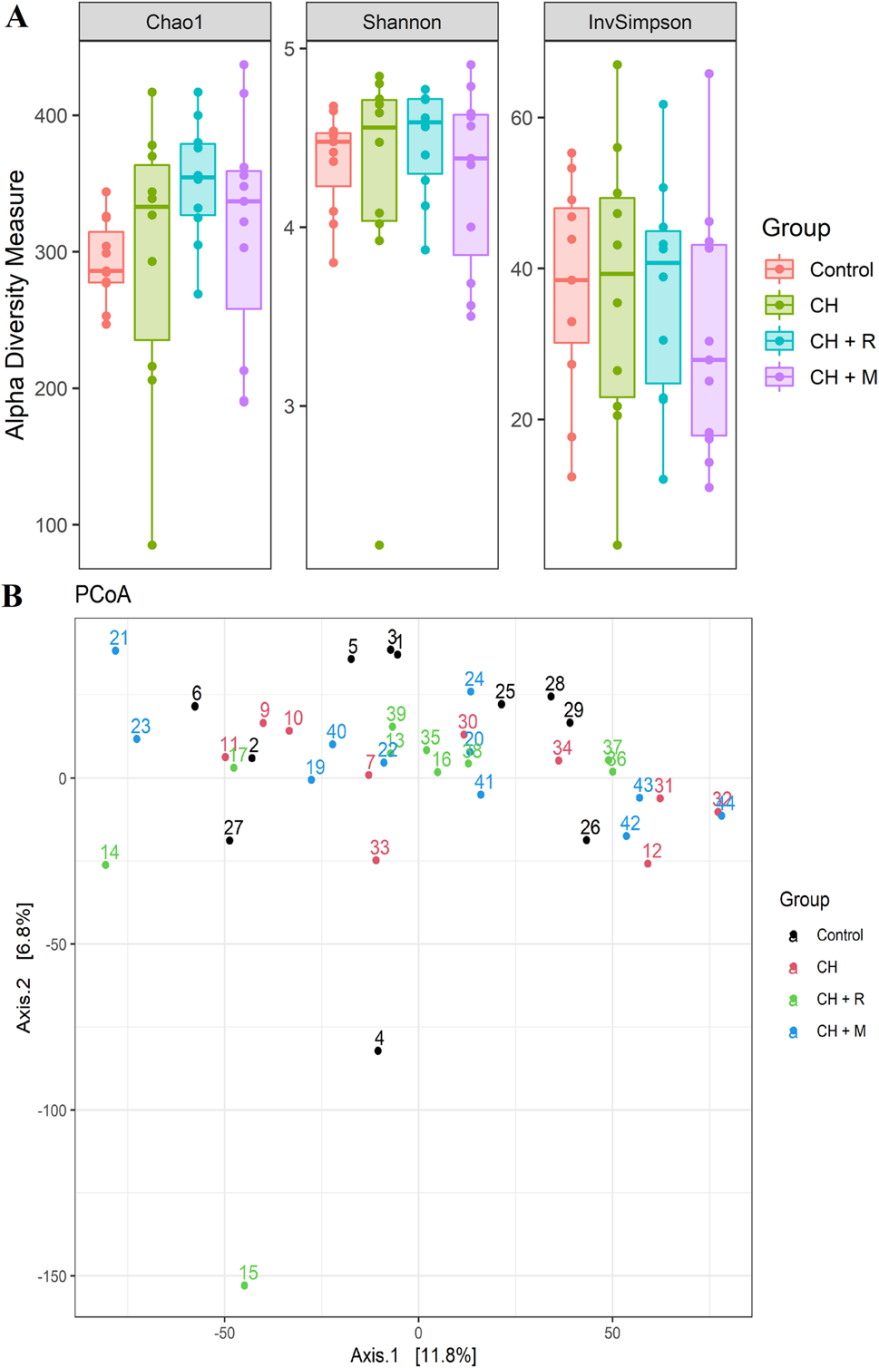
(**A**) Boxplots showing alpha diversity for Chao1, Shannon, InvSimpson indices. (**B**) PCoA plot using a Euclidian distance matrix. Dietary treatments: Control = control diet; CH = *Chlorella vulgaris* diet; CH + R = *C. vulgaris* diet supplemented with Rovabio® Excel AP; CH + M = *C. vulgaris* diet supplemented with a pre-selected enzymatic mixture.

For the alpha diversity, Chao1, Shannon and InvSimpson indices were calculated. None of the treatment influenced the alpha diversity measures (Fig. 1A).

For the beta diversity, meaning the differences in microbial composition between samples, a PCoA plot using a Euclidian distance matrix (calculated based on normalized row counts with the variance stabilization function of DESeq2—Table 5), was created (Fig. 1B). The plot does not show a clear separation of the samples based on treatment, but the results of the Adonis test indicate that diet significantly influenced the microbial composition (R^2^ = 0.09, P < 0.05). Although, the pair-wise Adonis test does not evidence any significant results for each of the possible comparisons. However, when the results for all CH-fed groups are combined and compared against the control group, there is a significant effect (R^2^ = 0.04, P < 0.05). In addition, the PERMDISP test was not significant, confirming the results of the Adonis test.

**Table 5:** DESeq2 output and contrast.

Contrast	ASVs^a^	baseMean^b^	log_2_FC	lfcSE^c^	P-value	Genus
CH + R vs. control	ASV537	6.84	5.69	1.34	0.00	*Rhodopseudomonas*
	ASV688	3.94	5.23	1.48	0.00	*Prosthecomicrobium*
	ASV149	90.0	2.13	0.54	0.00	*Colidextribacter*
	ASV8	1515	1.04	0.34	0.00	*Lactobacillus*
	ASV191	251	− 2.30	0.63	0.00	*Ruminococcus*
	ASV25	2136	− 2.65	0.76	0.00	*Treponema*
	ASV124	102	− 3.46	0.66	0.00	*Helicobacter*
	ASV446	14.7	− 4.46	1.51	0.00	*Asteroleplasma*
CH + M vs. control	ASV4	1631	6.87	1.44	0.00	*Escherichia/Shigella*
	ASV23	2322	2.04	0.66	0.00	*Alloprevotella*
	ASV108	330	1.86	0.63	0.00	*Sutterella*
	ASV149	90.0	1.75	0.53	0.00	*Colidextribacter*
	ASV199	51.6	− 1.26	0.42	0.00	*Solobacterium*
	ASV151	60.8	− 2.91	0.63	0.00	*Catenibacterium*
	ASV99	108	− 3.61	1.26	0.00	*Streptococcus*
	ASV446	14.7	− 4.48	1.48	0.00	*Asteroleplasma*
	ASV239	68.2	− 4.67	1.36	0.00	*Mitsuokella*
CH vs. control	ASV8	1515	1.17	0.34	0.00	*Lactobacillus*
	ASV108	330	2.56	0.64	0.00	*Sutterella*
	ASV149	90.0	2.06	0.54	0.00	*Colidextribacter*
	ASV537	6.84	6.50	1.33	0.00	*Rhodopseudomonas*
	ASV688	3.94	5.17	1.48	0.00	*Prosthecomicrobium*
CH vs. CH + R	ASV124	102	3.72	0.68	0.00	*Helicobacter*
CH + M vs. CH + R	ASV4	1631	− 7.67	1.48	0.00	*Escherichia/Shigella*
All (CH, CH + R, CH + M) vs. control	ASV8	1515	1.04	0.34	0.00	*Lactobacillus*
	ASV25	2136	− 2.65	0.76	0.00	*Treponema*
	ASV124	102	− 3.46	0.66	0.00	*Helicobacter*
	ASV149	90.0	2.13	0.54	0.00	*Colidextribacter*
	ASV191	251	− 2.30	0.63	0.00	*Ruminococcus*
	ASV446	14.7	− 4.46	1.51	0.00	*Asteroleplasma*
	ASV537	6.84	5.69	1.34	0.00	*Rhodopseudomonas*
	ASV688	3.94	5.23	1.48	0.00	*Prosthecomicrobium*

Control = control diet; CH = *Chlorella vulgaris* diet; CH + R = *C. vulgaris* diet supplemented with Rovabio® Excel AP; CH + M = *C. vulgaris* diet supplemented with a pre-selected enzymatic mixture.

^a^ASVs: abundant amplicon sequence variants.

^b^baseMean: mean of normalized counts of all samples.

^c^lfcSE: standard error estimated for the log2 fold change.

**Figure 2:**
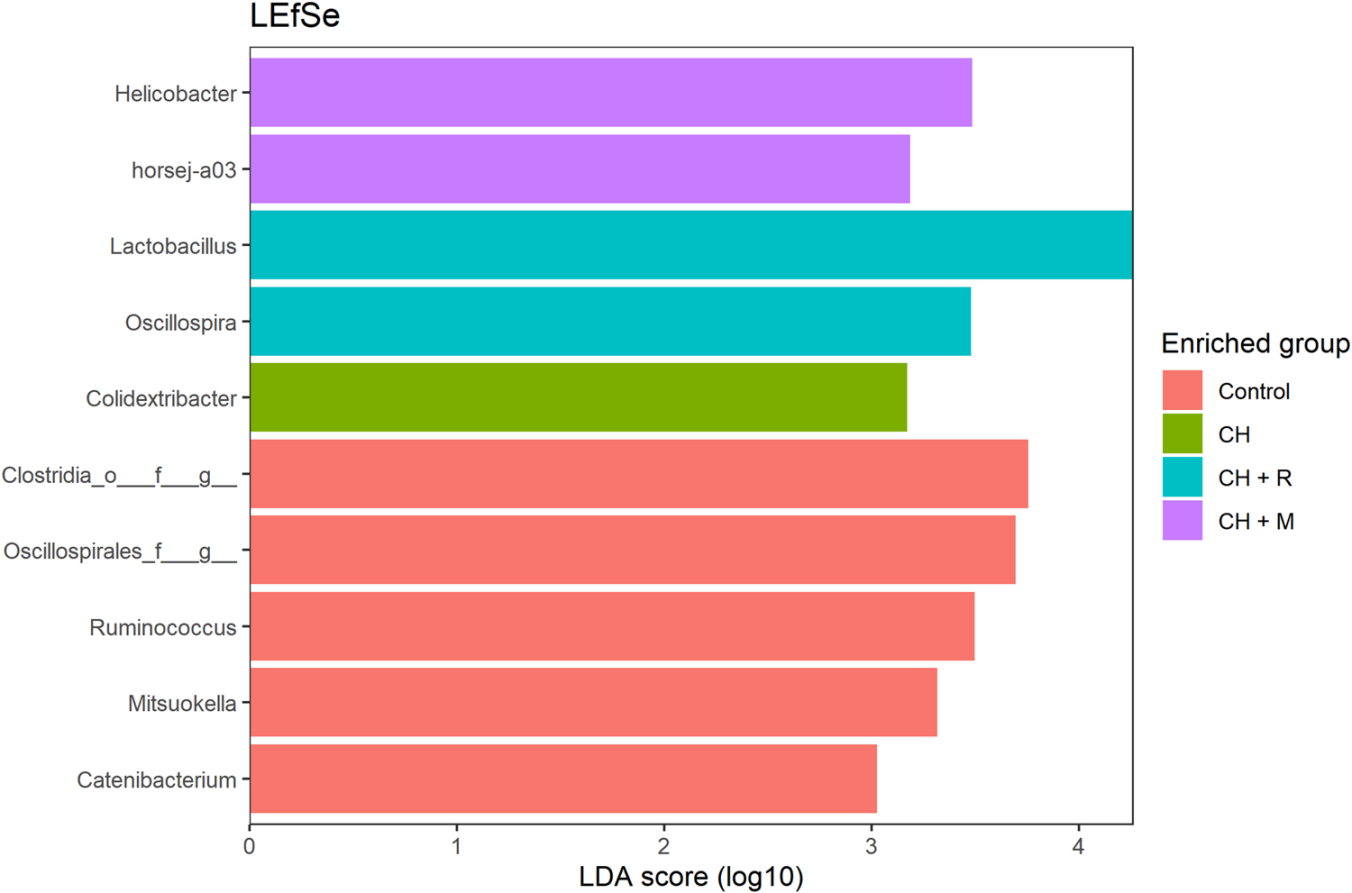
Barplot of Linear discriminant analysis (LDA) effect size (LEfSe). Horizontal bars represent the effect size for each taxon. The length of the bar represents the LDA score. LDA threshold score for discriminative features was set to 3.0. Dietary treatments: Control = control diet; CH = *Chlorella vulgaris* diet; CH + R = *C. vulgaris* diet supplemented with Rovabio® Excel AP; CH + M = *C. vulgaris* diet supplemented with a pre-selected enzymatic mixture.

In order to identify which taxa contributes to does differences, we used the LEfSe on the data aggregated at Genus level. The differential expressed Taxa are reported in Fig. 2.

As shown in Fig. 2, animals from the CH group had a higher abundance of a single bacterial taxa from genus *Colidextribacter*. This genus is constituted by a single species *Colidextribacter massiliensis*, which was isolated from human gut microbiota^8^. Overall, the relative abundance of this taxa is quite low (0.19 ± 0.14%). CH + R group had a higher abundance of genera *Oscillospira* and *Lactobacillus.* Whereas CH + M supplementation increased the abundance of bacteria from genus *Helicobacter* and *horsej*-a03 (family Oligosphaeraceae). Finally, animals from the control group were characterized by having bacteria belonging to *Ruminococcus*, *Mitsuokella*, *Catenibacterium* and some non-characterized bacteria belonging to Oscillospirales and Clostridia.

## Discussion

This study assessed the effect of dietary inclusion of 5% *C. vulgaris*, alone and in combination with 2 exogenous carbohydrase formulations, on recently weaned piglet performance, nutrient digestibility and intestine morphology, fermentation and microbiota. To the best of our knowledge, this is the first time that the subject was assessed in such detail in the newly weaned piglet. We established that the dietary inclusion of *C. vulgaris* as a feedstuff had no impact on growth performance of piglets, although ADFI was significantly higher in the groups fed with microalga. Such higher ADFI was not enough to significantly increase the growth rate of the animals. In the future, there is interest in confirming these results through a growth performance trial that involving a larger number of animals and ad libitum access to experimental diets. In addition, the supplementation of *C. vulgaris*-based diets with exogenous enzymes (Rovabio® Excel AP and the pre-selected 4-carbohydrase mixture) did not exert a particular influence on animal performance parameters of the piglets. Such results are in accordance with a performance study on growing-finishing pigs (59.1 to 101.9 kg), where the dietary inclusion of *C. vulgaris* and exogenous enzymes did not influence animal productive parameters^9^. Nevertheless, there are several studies on the subject that use *C. vulgaris* as a supplement (≤ 1% in diet) in piglet feeding to have a prebiotic effect^4,5^. For instance, Furbeyre et al*.*^4^ used 1% *C. vulgaris* in piglets’ diets to mitigate the post-weaning stress. These authors found no significant effects on ADFI, ADG and FCR. In addition, the same authors performed a trial with *C. vulgaris* via drinking water (385 mg/kg live weight) in weaning piglets with the same aim and also found no significant differences for ADFI, ADG and FCR^10^.

The TTAD of nutritional fractions was negatively affected by *C. vulgaris* incorporation, particularly the fibre fractions. This indicates a low efficiency of carbohydrase formulations in the *C. vulgaris* cell wall degradation in the intestine. However, the CH + R group had values closer to those of the control group than to those of the CH + M animals. Therefore, it could be speculated that the Rovabio® Excel AP has a higher *C. vulgaris* cell wall degradation ability than the 4-carbohydrase mixture. As Rovabio® Excel AP contains predominantly β-xylanases and β-glucanases, such hydrolytic activity was likely due to the degradation of the small amount of xylans and β-glucans in the *C. vulgaris* cell wall^11^. After all, it is noteworthy to mention that dietary treatment influenced the fibre profile that reached piglets’ large intestine. Furthermore, the TTAD of nutrients was significantly different for the 2 collection periods, with worse results for the second period. This indicates a difficulty of the piglets to adapt and digest diets containing high levels of *C. vulgaris*. Although no effects of diet and collection period on faecal consistency were observed, the higher value associated to animals fed with microalga in the second period seems to agree with the lower TTAD values determined.

The viscosity of duodenum plus jejunum contents slightly increased in the groups fed with microalga by comparison with the control group. Thus, it could be suggested that this increase in digesta viscosity did not result from the presence of soluble polysaccharides, such as arabinoxylans and β-glucans, as commonly observed in wheat or barley-based diets, since the presence of xylanases and β-glucanases in the CH + R group had no effect on viscosity. It is well known that higher digesta viscosity limits the access of endogenous enzymes to their target substrates^7^. However, this effect disappeared in the ileum content, where no differences in viscosity were found between experimental groups, and a compensatory small intestine enlargement was not found. Regarding the intestinal morphology, the incorporation of microalga in piglets’ diets affected only the duodenum villus height. This effect indicates the development of intestinal tissue in order to increase nutrients absorption. Similarly, Furbeyre et al*.*^4^ detected an increase in villus height in the jejunum, highlighting the positive effect of *C. vulgaris* supplementation on mucosal restoration or development after weaning. In our study, the increase of duodenum villus height seems to be able to compensate, at least in part, the higher digesta viscosity promoted by the *C. vulgaris* feed incorporation.

Regarding VFA concentration in the caecum, the CH + M group showed a significantly lower quantity of total VFA compared with all other groups. The lower degradation rate reported in this group may be associated with the lower quantity of cell wall material that reach the caecum. This aspect suggests a possible effect of the enzymatic mixture on the degradation of microalga cell walls at the level of small intestine. In the colon, the concentration of total VFA was decreased for all animals fed with the microalga. This decrease indicates a low level of fermentable carbohydrates in the colon of piglets fed with *C. vulgaris*, with the consequent change in the microbial fermentation profile. The recalcitrant cell wall may justify the presence of less fermentable cell wall constituents in this digestive compartment. Several studies have associated insoluble dietary fibre content of diets to the effect on fermentation, generation and absorption of VFA at the level of the large intestine^12^. Thus, the lower TTAD of fibre fractions of the CH + M group could also explain the lower VFA values. Additionally, Montoya et al*.*^13^ refer the importance of not extrapolating the results because the type of dietary fibre influences the quantity of VFA produced by fermentation. Information on physical characteristics, molecular structure and chemical composition of the fibre of *C. vulgaris* microalga is scarce and, therefore, further research has still to be conducted on this aspect.

Microbiome results suggest that the use of *C. vulgaris* in piglet feeding, in combination or not with enzymes, significantly affects the faecal bacterial structure of piglets, as previously observed in humans^14^. In addition, C. vulgaris incorporation supplemented with 0.005% Rovabio increased the relative abundance of *Lactobacillus* and *Oscillospira*. *Lactobacillus* which is one of the most represented genera. This bacterial taxon usually constitutes the core member of a healthy pig microbiota^15^, preventing intestinal colonization of enteric pathogens^16^. Oscillospira is an anaerobic bacterial genus from the Clostridial cluster, that is widely studied in human research due to its role in preventing specific diseases, such as obesity-related metabolic diseases^17^. In addition, due to its ability to produce short-chain fatty acids (SCFAs) such as butyrate, it has been proposed as a potential probiotic^18^. On the other end, the contrast highlighted that the CH + M group was characterised by*Helicobacter* genus, compared to the other groups. The highest prevalence of *Helicobacter*, together with the highest pH, in the upper part of the small intestine should be ascribed to the effect of enzyme mixture on the degradation of microalgae cell wall, thus providing a substrate for proteolytic bacteria. It thus seems that the efficacy of this enzyme mixture was site dependent. Indeed, the TTAD is lower, compared with that of the other CH groups, especially for the fibrous fractions. In accordance, VFA concentrations are lower in the CH + M group than in the other groups, particularly in the cecum. This suggests a lower efficiency of the 4-enzyme mixture to degrade microalga cell wall. In addition, *C. vulgaris* supplementation reduced the abundance of Ruminicoccus, a bacterial taxon known for its fibrinolytic activity and the production of VFA, especially butyrate^19^, which can also explain the lower VFA and TTAD of fibrous fractions.

## Conclusion

In this study, we showed that the inclusion of 5% *C. vulgaris* in the diet improves piglets’ gut health without compromising animal performance. Data indicates that although nutrients digestibility, mainly for fibre fractions, decreases by the incorporation of microalga in the diet, production performance of piglets is not impaired. This is likely explained by two compensatory mechanisms, the gut mucosa development and the probiotic properties of some specific bacterial taxa in the intestine (*Colidextribacter*, *Oscillospira* and *Lactobacillus*).

Moreover, the dietary supplementation with exogenous carbohydrases does not seem to be necessary for feeding piglets with *C. vulgaris*-based diets at this level of incorporation.

Considering that weaning is a critical period for piglets’ health, the inclusion of *C. vulgaris* as a prebiotic and sustainable feed ingredient in the diet is an interesting strategy for swine production, particularly for the recently weaned piglet. However, its cost-effective utilization for this purpose warrants further investigation.

## Methods

### Experimental design, diets and animal performance

Following the principles and specific guidelines of the European Union legislation (2010/63/EU Directive), as well as the ARRIVE guidelines 2.0 (https://arriveguidelines.org/arrive-guidelines), all the procedures used in this animal experiment were revised by the Ethics Commission of ISA and accepted by the Animal Care Committee of the National Veterinary Authority (Process Number 0421/2017, Direção Geral de Alimentação e Veterinária, Portugal).

Forty-four post-weaned piglets (50% Pietrain × 25% Large White × 25% Landrace), weaned at 28 d of age and with an initial live weight of 11.2 ± 0.46 kg (mean ± SD) were obtained from a commercial farm. Each animal was allocated to a crate equipped with a feeder, a stainless-steel nipple, a heating lamp and plates for separation of faeces and urines. The environmental conditions of the room were the same as described previously^20^. Animals had 2 days for environmental adaptation and stabilization of stress and digestive condition. After this period, 2 animals failed to this adaption and were withdrawn from the study. Each animal had access to one of the 4 experimental diets: (1) cereal and soybean meal-based diet (control, n = 11); (2) control diet with 5% of *C. vulgaris* (CH, n = 10); (3) control diet with 5% of CH supplemented with 0.005% of Rovabio® Excel AP (Adisseo, Antony, France) (CH + R, n = 10); and (4) control diet with 5% CH supplemented with 0.01% of the 4-carbohydrase mixture described by Coelho et al*.*^9,21^ (CH + M, n = 11). The microalga was supplied by the company Allmicroalgae—Natural Products SA, Pataias, Portugal as freeze-dried powder and included as supplied in the diets. Its chemical composition was previously described by our team in Coelho et al*.*^9^. The detailed description of the experimental diets is presented in Supplementary Table S2.

Piglets were fed daily, with the same amount of feed provided per animal. To calculate ADFI, feed refusals were recorded daily. Animals had ad libitum access to water. Moreover, the individual body weight was recorded weekly in order to calculate ADG and FCR. The faeces were collected for 2 periods of 6 d in order to calculate TTAD. The following equation: TTAD = ((N_in_ − No_ut_)/N_in_) × 100 was used. N_in_ represents the total intake of a specific nutrient in the feed and N_out_ represents the total faecal output for the same nutrient. In addition, the consistency of the faeces was recorded daily, according to the following scale: 0 (normal), 1 (soft faeces) or 2 (diarrhoea).

### Slaughtering and sampling

After 21-d experimental time all animals were slaughtered using electrical stunning followed by exsanguination. The gastrointestinal tract was removed to measure the length of the small and large intestines. Also, the contents of stomach, duodenum plus jejunum, ileum, caecum and colon were collected, immediately analysed (pH and viscosity determinations) or stored at – 20 °C for VFA determination. For histological analysis, 3 segments of the small intestine were collected: duodenum (10 cm below pylorus), jejunum (5.5 m below pylorus) and ileum (60 cm above ileum-caecal valve). These tissue samples were fixed into 10% buffered formalin solution and then processed for paraffin embedding. For microbiome analysis, faecal samples were collected and stored at − 80 °C until DNA extraction.

### Chemical analysis of diets and faeces

All the methods used for diets and faeces analysis were previously described^7^. Briefly, faecal samples were dried at 60 °C for 72 h in an oven with ventilation. Diets and dried faecal samples were ground in 1 mm diameter mesh mill and analysed, in duplicate, for DM, ash, CP and CF contents, following the methods described by AOAC^22^. NDF, ADF and acid detergent lignin (ADL) were performed sequentially using crucibles system by Van Soest et al*.*^23^. Hemicellulose and cellulose were calculated as NDF-ADF and ADF-ADL, respectively.

Determination of amino acids, fatty acid methyl esters (FAME), diterpene profile, pigments and mineral composition were performed in the microalga and diets. The amino acids, except tryptophan, were measured as described in Commission Regulation (EC) No 152/2009^24^. Briefly, cysteine and methionine were oxidised to cysteic acid and methionine sulphone, respectively, prior to hydrolysis. All the other amino acids, except tryptophan, were determined in hydrolysates of unoxidized samples. The determination of tryptophan in samples was performed according to la Cour et al*.*^25^. All amino acids were analysed by HPLC (Agilent 1100, Agilent Technologies, Avondale, PA, USA), combined with automated pre-column derivatisation using o-phthaldialdehyde and 9-fluorenylmethyl chloroformate, as reported by Henderson et al*.*^26^. FAME were analysed by extraction and acid transesterification, using fatty acid 21:0 as the internal standard^27^. Diterpene profile was conducted by a single n-hexane extraction succeeded by HPLC^28^. The determination of pigments was performed according to Teimouri et al*.*^29^, with small modifications. After overnight extraction with acetone, obtained solutions were centrifuged at 2000×*g* for 5 min and analysed by UV–Vis spectrophotometry measuring the absorbance at different wavelengths (Ultrospec 3100 pro, Amersham Biosciences, Little Chalfont, UK). Pigment contents were calculated using the equations described by Hynstova et al*.*^30^. The mineral composition was performed following the previously described protocol^31^.

The detailed chemical composition of the microalga and experimental diets is shown in Table 6.

**Table 6:** Chemical composition of *Chlorella vulgaris* microalga and experimental diets.

	Microalga powder	Diets			
		Control	CH	CH + R	CH + M
**Proximate composition, g/100 g (as fed basis)**					
DM	93.1	90.5	90.8	90.8	90.9
OM	81.3	85.1	85.2	85.3	85.3
Ash	11.8	5.43	5.65	5.47	5.60
CP	42.8	19.3	19.2	19.5	19.4
NDF	32.2	12.9	11.9	12.9	10.4
ADF	19.4	2.76	2.45	2.58	2.54
CF	8.75	5.29	5.39	5.39	5.63
**Limiting amino acids, g/100 g (as fed basis)**					
Cysteine	0.297	0.275	0.242	0.225	0.227
Lysine	3.87	1.32	1.59	1.59	1.58
Methionine	0.819	0.318	0.356	0.335	0.322
Threonine	2.23	0.95	0.88	0.90	0.97
Tryptophan	0.895	0.353	0.357	0.337	0.350
**Fatty acid composition, % total fatty acids**					
14:0	1.13	0.351	0.380	0.380	0.361
16:0	17.2	10.6	11.0	10.9	11.1
16:1c9	3.90	0.158	0.903	0.900	0.677
17:0	0.234	0.095	0.104	0.103	0.104
17:1c9	0.610	0.040	0.583	0.643	0.828
18:0	3.00	3.35	3.33	3.38	3.26
18:1c9	11.7	24.8	24.5	24.5	24.6
18:1c11	–	0.909	1.16	1.16	1.10
18:2n-6	11.2	55.8	53.3	53.2	52.9
18:3n-3	10.1	1.55	2.18	2.23	2.52
20:0	0.174	0.306	0.286	0.286	0.283
20:1c11	0.127	0.227	0.224	0.197	0.215
22:0	0.060	0.566	0.531	0.542	0.521
22:1n-9	–	0.105	0.108	0.104	0.085
**Pigments, µg/g**					
Chlorophyll-a^a^	906	3.38	109	130	135
Chlorophyll-b^b^	171	6.05	31.9	42.6	39.8
Total chlorophylls^c^	1077	9.43	141	172	174
Total carotenoids^d^	228	2.67	36.9	44.5	52.9
Total chlorophylls and total carotenoids^e^	1305	12.1	178	217	227
**Diterpene profile, µg/g**					
β-Carotene	198	–	13.3	13.7	14.5
α-Tocopherol	19.2	28.6	19.9	22.1	24.2
β-Tocopherol	0.339	1.11	1.10	1.00	1.12
γ-Tocopherol	0.521	2.52	2.00	2.21	2.11
δ-Tocopherol	0.371	0.502	0.334	0.387	0.396
α-Tocotrienol	–	3.43	3.73	3.58	3.53
γ-Tocotrienol	0.560	1.38	1.51	1.69	1.46
**Macrominerals, g/100 g (as feed basis)**					
Calcium	0.703	0.666	0.837	0.785	0.824
Phosphorus	2.04	5.19	6.42	5.94	5.36
Potassium	2.92	1.25	1.33	1.29	1.16
Sodium	0.382	1.53	1.84	1.55	2.04

Control = control diet; CH = *Chlorella vulgaris* diet; CH + R = *C. vulgaris* diet supplemented with Rovabio® Excel AP; CH + M = *C. vulgaris* diet supplemented with a pre-selected enzymatic mixture.

*DM* dry matter, *OM* organic matter, *CP* crude protein, *NDF* neutral detergent fibre, *ADF* acid detergent fibre, *CF* crude fat.

^a^Chlorophyll-a (Ca) = 11.24 × A662 nm − 2.04 × A645 nm.

^b^Chlorophyll-b (Cb) = 20.13 × A645 nm − 4.19 × A662 nm.

^c^Total chlorophylls (Ca + Cb) = 7.05 × A662 nm + 18.09 × A645 nm.

^d^Total carotenoids (Cx + Cc) = (1000 × A470 nm − 1.90 × Ca − 63.14 × Cb)/214.

^e^Total chlorophylls and carotenoids = (Ca + b) + (Cx + c).

### Gut content analysis

The pH measurement of the contents of stomach, duodenum plus jejunum, ileum, caecum and colon were immediately determined, using a glass electrode pH meter (Metrohm 744, Metrohm AG, Herisau, Switzerland). The viscosity of small intestine contents was measured as previously described^7^.

Caecum and colon contents (5 g) were collected in a 5% (v/v) o-phosphoric acid until the quantification of the following VFA: C2, C3, C4, C5 and. These compounds were quantified by gas chromatography as previously described^32^, on the supernatant of thawed samples centrifuged at 8000×*g* for 10 min. The 4-Methyl valeric acid was used as internal standard.

### Gut histological analysis

Microscopic examination and measurement of villi heights and widths and crypt depths were performed in 7 μm thick tissue sections, stained with haematoxylin–eosin. An Olympus BX 51 microscope equipped with 4× and 10× lenses was used. Images were digitally captured with an Olympus DP 21 camera. The height and width of the villi and the depth of the crypts were measured using the Olympus DP-Soft software. Ten intact and correctly oriented villi and crypts from each intestinal region were selected for each piglet.

### Gut microbiota analysis

Total bacterial DNA was isolated and extracted with QIAamp® Fast DNA Stool Mini Kit (Qiagen, Hilden, Germany) following the manufacturer’s instructions. DNA concentration and purity (absorbance ratio 260/280 and 260/230) of the isolated DNA were checked by spectrophotometry on the NanoDrop (Fisher Scientific, 13 Schwerte, Germany).

For the microbiological analysis of faecal samples, the V4 region of the 16S rRNA gene (~ 380 bp) was amplified 515f: 5′‐GTGYCAGCMGCCGCGGTAA‐3′; 806r: 5′‐GGACTACNVGGGTWTCTAAT‐3′ and sequenced using the Illumina 2 × 250 bp MiSeq platform (Illumina Inc., San Diego, CA, USA)^33^.

The bioinformatic analysis was performed using DADA2 1.14.0^34^ running on R 4.0.2. For the taxonomic assignment, the SILVA database release 138 was used as reference^35^.

### Statistical analysis

Data homogeneity and normality were verified. Growth performance, nutrient digestibility, intestinal morphology and VFA data were analysed using the PROC MIXED of SAS software package (version 9.4; SAS Institute Inc., Cary, NC, USA). The consideration of the model was the dietary treatment as single effect and the piglet as experimental unit. When significant effects of treatments were detected, least square means were compared using the PDIFF with Tukey–Kramer adjustment options of SAS. TTAD data were analysed using the PROC MIXED, considering repeated measures over time to test the effect of diet, period and their interaction. Results were considered significantly different when the P-value < 0.05.

Regarding the statistical analysis of the microbiome, data on alpha diversity, beta diversity and taxonomic composition were carried in R 4.0.2 using phyloseq^36^, Vegan^37^ and DESeq2^38^ packages. To test the differences between the groups for the alpha diversity, a Multifactorial ANOVA (MANOVA) model was fitted, considering sequencing depth and group as factors. For the beta diversity, the Euclidian distance was calculated, and the differences between groups were tested using a non-parametric PERMANOVA (Adonis) model, with 999 permutations, pair-wise contrast were made using the pairwise Adonis function provided by the pairwise Adonis R package^39^. In addition, to tests the homogeneity of dispersion among them a PERMDISP test was used^40^. Samples abundances were normalized using variance stabilizing transformation provided by DESeq2 package. Differences for the taxonomic composition between treatments were tested using Linear discriminant analysis (LDA) effect size (LEfSe) aggregating the data at Genus level, LDA score cut-off of 3 was used to discriminate bacterial taxa^41^. The P-values were adjusted for multiple comparison using the False Discovery Rate (FDR) method. Significance was declared if P-value < 0.05 and a trend was considered when 0.05 < P-value < 0.10.

## Supplementary Information

Supplementary Figure S1.

Supplementary Figure S2.

Supplementary Table S1.

Supplementary Table S2.

## Data Availability

Data generated or analysed during this study are included and summarized in this published article and its Supplementary files. Raw data will be supplied upon reasonable request.
