# Supplementary figures and images for: Influence of Chlorella vulgaris on growth, digestibility and gut morphology and microbiota of weaned piglet
Source: Sci Rep. 2022 Apr 9;12:6012. doi: 10.1038/s41598-022-10059-5 (PMC8994764; doi:10.1038/s41598-022-10059-5)

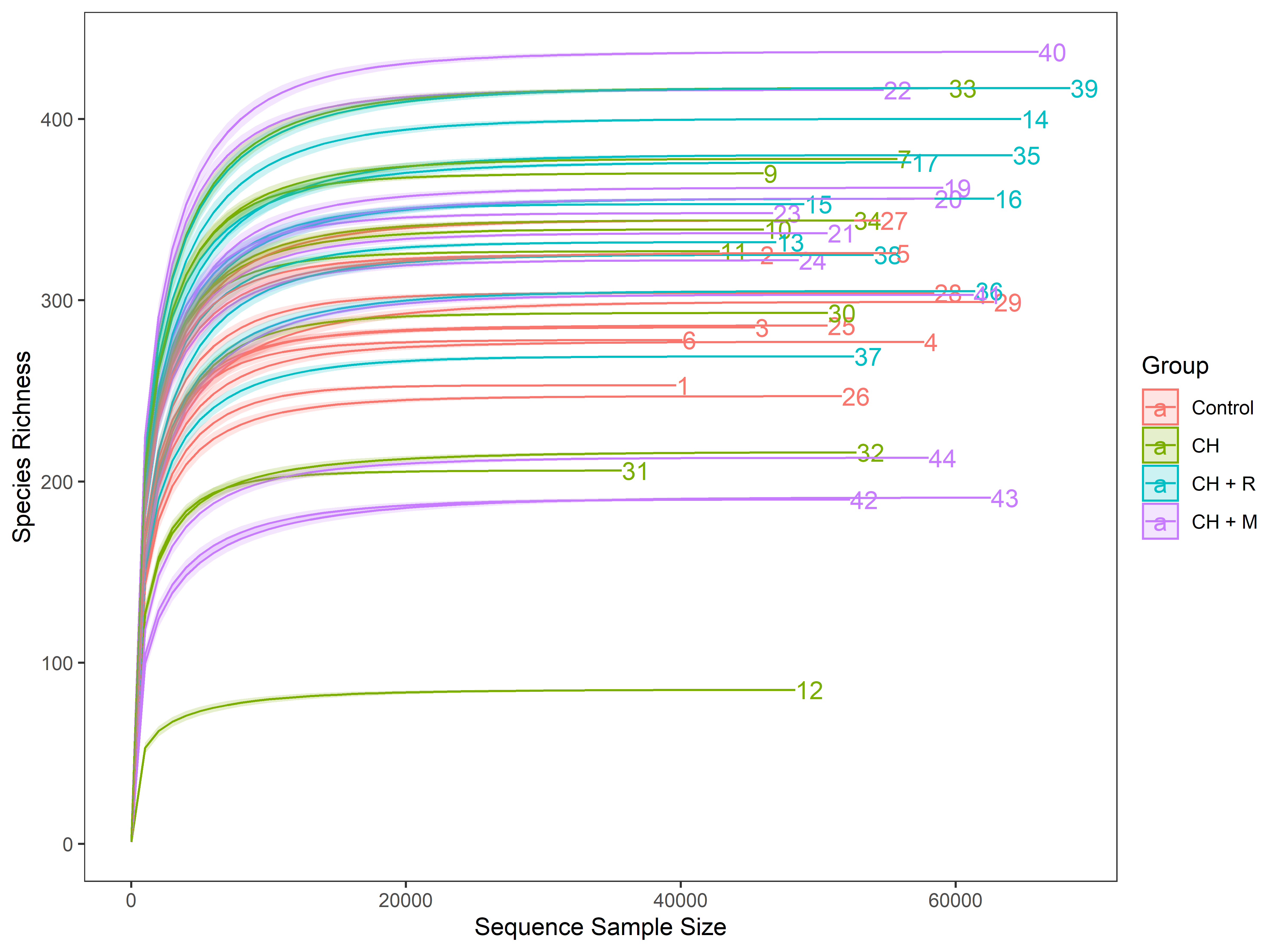

Supplement: Supplementary file 1 — Supplementary Figure S1. [file 41598_2022_10059_MOESM1_ESM.tiff]
